# Crystal structure of the MyRF ICA domain with its upstream β-helical stalk reveals the molecular mechanisms underlying its trimerization and self-cleavage

**DOI:** 10.7150/ijbs.57673

**Published:** 2021-07-13

**Authors:** Pei Wu, Xiangkai Zhen, Bowen Li, Qian Yu, Xiaochen Huang, Ning Shi

**Affiliations:** 1 State Key Laboratory of Structural Chemistry, Fujian Institute of Research on the Structure of Matter, Chinese Academy of Sciences, Fujian College, University of Chinese Academy of Sciences, 155 Yangqiao Road West, Fuzhou, 350002, China; 2 Institute of Vascular Anomalies, Shanghai TCM-Integrated Hospital, Shanghai University of Traditional Chinese Medicine, 230 Baoding Road, Hongkou, Shanghai, 200082, China; 3 University of Chinese Academy of Sciences, Beijing, 100049, China

**Keywords:** membrane transcription factor, Myelin gene regulatory factor (MyRF), intramolecular chaperone autocleavage (ICA) domain, triple-beta-helix, crystal structure

## Abstract

Myelin gene regulatory factor (MyRF), a novel membrane transcription factor expressed on the endoplasmic reticulum membrane, functions as a trimer. The trimerization of MyRF is associated with a fragment between the DNA binding domain and transmembrane domain that shares homology with the triple-β-helix and intramolecular chaperone autocleavage (ICA) domain of phage tailspike proteins. The molecular details of these domains in eukaryotes have not been elucidated. Here, we present the crystal structure of the MyRF ICA domain with its upstream β-helical stalk, determined at 2.4Å resolution. The structure showed that its upstream β-helical stalk is different from the triple β-helix reported before. This is the first structure of the mammalian protein with a triple β-helix. Structure analysis demonstrated that the triple α-helical coiled-coil formed at the MyRF ICA domain C-terminal was the main driving force for the trimerization. Additionally, our findings showed that MyRF was cleaved via a highly conserved serine-lysine catalytic dyad mechanism and that cleavage would be activated only if the ICA domains were organized as trimers. In contrast to the viral ICA domain, almost no interaction was found between the MyRF ICA domain and its upstream neighboring β-helix of the stalk; thus, activation of self-cleavage may not be triggered by the upstream region of the ICA domain, contrary to the observations made in phages. These findings provided an important insight into the molecular mechanisms of MyRF trimerization and self-cleavage.

## Introduction

Most cell activities are controlled by the expression of transcription factors. Some transcription factors are initially synthesized as precursors of the membrane protein and sequestered in the membranes of cells or organelles in eukaryotic cells. Only after cleavage and liberation from the membrane can they enter the nucleus to accomplish gene transcription and regulatory functions. Cleavage and release of the sequestrated membrane transcription factors under control is a common mechanism for the regulation of gene expression. Three types of membrane transcription factors have been reported according to the enzymes involved in their cleavage. The first type of membrane transcription factor is cleaved by the Golgi site-1 and site-2 proteases (S1P and S2P) in the intramembrane (regulated intramembrane proteolysis, RIP). The sterol response element-binding protein is a typical member of this type of membrane transcription factors 1. The second type of membrane transcription factor is cleaved by presenilin-dependent regulated intramembrane proteolysis (PS-RIP) 2, 3. One of the current members of this type is the intracellular domain of the Notch receptor, which is released from the cell membrane to participate in the transcriptional activation of target genes 4, 5. The third type of membrane transcription factor is cleaved by the ubiquitin/proteasome-dependent pathway 6. Members of this type include the yeast membrane transcription factors SPT23 and MGA2, distant homologs of the soluble cytosolic nuclear factor (NF)-κB/Rel of higher eukaryotes. These proteins are cleaved outside the transmembrane domain for release from the membrane. Their cleavage mechanism is similar to that of the precursor of the NF-κB transcription factor which is processed by proteasomes.

Recently, a new type of membrane transcription factor, i.e., myelin gene regulatory factor (MyRF), was found to exhibit a novel cleavage mechanism 7. This factor is initially expressed on the endoplasmic reticulum (ER) membrane with a large cytoplasmic N-terminal fragment, including a DNA binding domain (DBD) that can be released from the ER membrane. Studies have revealed that this release is caused by an autocatalytic self-cleavage reaction attributed to a fragment between the DBD and transmembrane domain, which is homologous to the intramolecular chaperone autocleavage (ICA) domain in bacteriophage endosialidases 8, 9. Functional assays have shown that this domain is essential for MyRF transcriptional activity 7, 10.

MyRF is expressed in oligodendrocytes as a key transcription factor in the formation and maintenance of myelination in the central nervous system 11. Moreover, this protein is highly expressed in several other tissues and plays important roles in other organs 12-14. Whole- genome sequencing studies have found that some MyRF mutants are associated with birth defects and abnormal development of the heart, genitourinary tract, diaphragm, lung, and eye 15-18. Some of these disease-related mutants are located on the ICA domain.

Previously, homologs of the ICA domain have been found only in diverse phages which can be classified into four different protein families: endosialidases (e.g., endoNF), K5 lyases (e.g., ElmA), the L-shaped tail fibers of coliphage T5 (e.g., LTF), and the neck appendage protein (e.g., GP12 of *Bacillus* phage GA-1). These proteins facilitate the assembly of N-terminal domains into a trimer 8. Once this trimer is folded correctly such that the key residues for the cleavage reaction enter into the proper positions, autocatalytic self-cleavage is triggered and the ICA domain is released, leaving a kinetically stable trimeric protein with triple-beta-helix 8, 9. Mutations that disrupt the self-cleavage or trimerization of the ICA / triple-beta-helix domain can prevent the formation of the functional N-terminal oligomers.

The MyRF ICA domain with its upstream β-helical stalk seems to have a similar function for driving MyRF to form a homotrimer and release its N-terminal fragments as a free transcription factor from the ER membrane by self-proteolysis. Although the biochemical functions of the triple-β-helix and ICA domain have been well-characterized in viral proteins, their homologies have not yet been described in membrane transcription factors. Moreover, because their homological sequences are extremely distant from those of viral proteins, the exact auto-cleavage mechanism of MyRF may differ; further studies are needed to assess these mechanisms.

Accordingly, in this study, we present the crystal structure of the MyRF ICA domain with its upstream β-helical stalk. To the best of our knowledge, this is the first structure of a mammalian protein with a triple-β-helix and ICA domain. Overall, our findings provide important insights into the molecular mechanisms underlying the homo-trimerization and autocleavage of MyRF.

## Results

### Structure of the MyRF ICA domain prior to cleavage

First, the mouse MyRF DNA fragment (residues 351-717) was cloned and expressed. The expressed protein containing DBD and ICA domains underwent autoproteolytic cleavage to produce two fragments. To gain insight into the folding process of the homotrimer and the details of the autoproteolytic cleavage reaction of MyRF, a precleavaged protein was generated by expressing a noncleavable mutant of the fragment (MyRFS587A) based on previous reports. The noncleavable MyRF_S587A_ protein was expressed and purified. Crystallization trials were performed using the final purified protein, and several crystals were obtained. We collected X-ray diffraction data from SSRF. The crystals belonged to the P321 space group. The space group parameters of some crystals were similar to those of the previously obtained DBD crystal, and the structure was solved by molecular replacement using the structure of MyRF DBD as a search model. Unfortunately, the solved structures contained only the DBD domain. The space group parameters of some other crystals were different from those of the DBD crystal, and the calculated cell volume was too small to accommodate the DBD molecule. Finally, the structure of this type of crystals was solved by single isomorphous replacement, including anomalous scattering using a selenomethionine (SeMet)-derivatized protein crystal.

**Figure 1:**
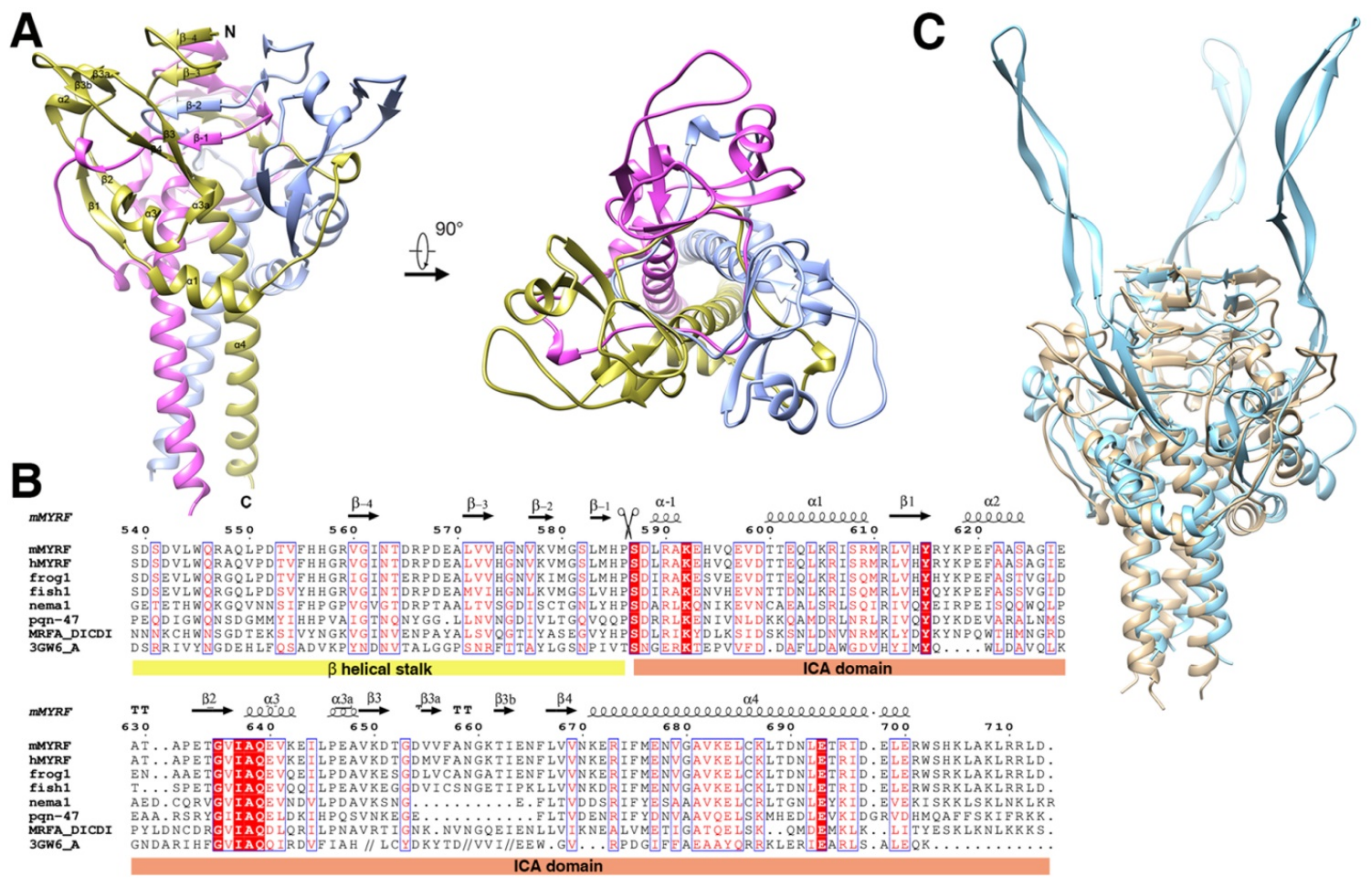
Structure of the homotrimer (crystallographic symmetry-related protomers) of the pre-cleavage MyRF ICA domain with its upstream β-helical stalk. All the strands and helix are labeled according to the structure of phage endoNF (PDB code: 3GW6). **A.** Overall side view and top view of the cartoon diagram. The three subunits in the MyRF trimer are colored in gold, ice-blue, and pink respectively. **B.** Secondary structure and sequence alignment of the MyRF ICA domain with the stalk. Mouse MyRF (539-713) is aligned with sequences from other *vertebrates* (human, frog, and fish), *nematode1, nematode2* (PQN47)), *Dictyostelium* (MrfA), and *phage* (endoNF). Secondary structures of the MyRF ICA domain are indicated on the top. The residue numbering along the top refers to the mouse MyRF. The alignment was performed by CLUSTALW 37, then edited and displayed with ESPript 38. The scissor indicates the cleavage site. White characters in red boxes indicate identity and red characters in white boxes indicate homologous residues. **C.** The superimposed structures of the trimer of the MyRF ICA domain with the stalk and the corresponding portion of endoNF_ (_PDB code:3GW6) colored in ice blue and tan respectively. The software Chimera was used to superimpose the structures and generate the figure.

The final structure was refined to 2.4 Å (Table 1) and consisted of two parts, the β-helical stalk and ICA domain. The space group was *P321* (a = 78.7 Å, b =78.7 Å, c = 138.5 Å, α=90.0º, β=90.0º, and γ=120.0º) and each asymmetric unit contained three monomers, which belonged to three different homotrimers. A crystal symmetry operation was used to generate the other two monomers of functional trimeric proteins. The overall structure of the trimer showed a drumstick-like outline with the stalk sitting on top of the trimer connecting the DBD and ICA domain. Each ICA monomer displayed an α1-β1-α2-β2-α3-β3-β3a-β3b-β4-α4 fold architecture (Figure 1A, 1B, and Figure S1). The C terminal α helix (α4) of each subunit of the trimer wrapped into left-handed superhelix and created a cylinder that was approximately 27 Å wide and 51 Å long. The short α1 and α3 helix surrounded the central cylinder in an almost perpendicular position.

The topology of the precleavage protein and the structure of the trimer were similar to those of the precleavage endoNF_S911A_ (phage endosialidase protein, PDB:3GW6) and the overall root mean squared deviation of the main chain was 2.05 Å (evaluating superpositions across all 98 fully populated columns in the final alignment; Figure 1C). The most prominent difference was that the antiparallel β-strands of β3-β3a-β3b-β4 which formed an extremely long tentacle and interacted with the triple-β-helix stalk and the functional domain upstream of the ICA domain in endoNF_S911A_, while formed a “donut” with the antiparallel β-strands of β1-α2-β2 in MyRF (Figure 3A) and did not interact with the upstream neighboring β-helix of the stalk at the top. Hydrogen bonds were formed between the helix residues E620, S624 (between β1 and β2) and the loop residues A658, V656 (between β3a and β3b) (Figure S2). The “donut” formed with four β-strands (β1-4) showed an outer diameter of approximately 25 Å and an inner diameter approximately 9 Å. A “cable” from the neighboring subunit threaded through the middle hole of the “donut”, and the cleavage site was located directly on this “cable” (Figure 3A).

### The β-helical stalk

**Figure 2:**
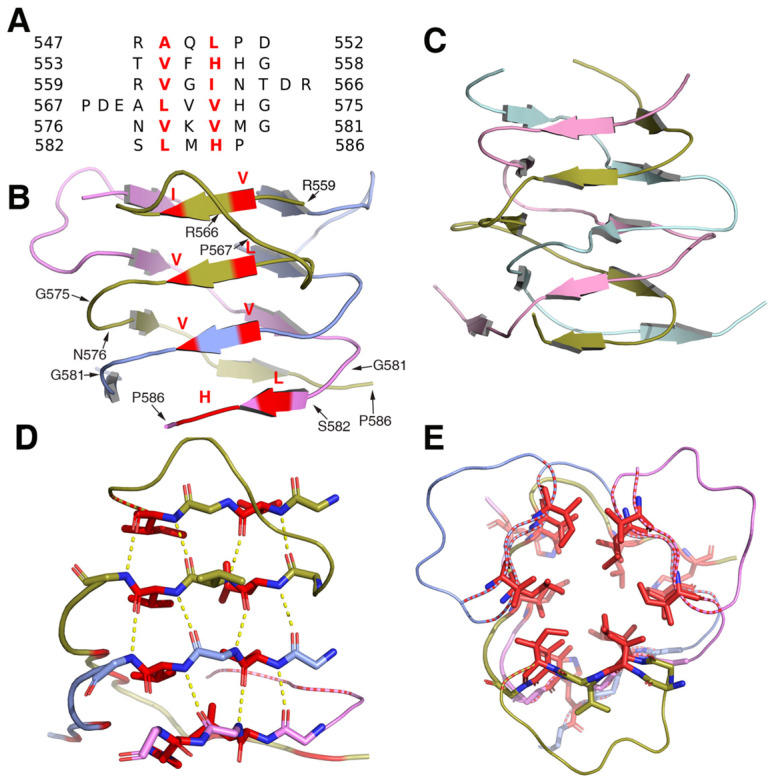
The structure of the homotrimer of the MyRF β-helical stalk. **A.** Structure-based sequence alignments of β-sheet elements within the MyRF β-helical stalk. The residues with the central side-chain stacks are colored red. **B.** Cartoon diagram of the homotrimer of the MyRF β-helical stalk showed a triangular β-sheet prism with a partially intertwined structure. The residues with the central side-chain stacks are colored red. **C.** The cartoon diagram of the homotrimer of the typical triple-β-helix of endoNF showed a fully intertwined structure. **D.** Stacks of four hexapeptide repeats from one side of a subunit are represented as sticks. The main-chain hydrogen bonds are shown in yellow dashed lines and the two residues with the central side-chain stacks are shown in red.** E.** The four contiguous stacks of the MyRF β-helical stalk homotrimer in the axial view are shown forming a triangular prism. The two residues with the central side-chain stacks are represented as red sticks.

Structural analysis showed that the β-helical stalk of MyRF formed a triangle prism with three β-sheets formed on each side by 4-6 parallel β strands, which came from three different subunits.The strands intertwined partially since the top of the two strands of the structure (residues 560-562 and 571-573) on the same side were from the same subunit (Figure 2A, 2B), in contrast to the typical triple-β-helix, in which all the β strands were fully intertwined, with no two neighboring β-strands on the same side of the same subunit (Figure 2C). The MyRF sequence included five additional residues between the top two strands, which allowed the β strands to wind back on the same side (Figure 2A). Hydrogen bonds were formed between the main chain of the β-strands in the sheets on each side (Figure 2D). The hydrophobic core of the stalk was filled with six residues in each layer, along with mixed stacks of Val, Leu, and His. Residues participating in core stacks were located at each β-strand with one residue apart (Figure 2E). In this configuration, the β-helical stalk of the MyRF was not as stable as the typical triple-β-helix (the calculated free energy of dissociation using the PISA program was 33.4 versus 69.4 kcal/mol). In addition, the MyRF stalk could not act as a sensor for trimerization to trigger the cleavage because there were almost no interactions between the MyRF stalk and the ICA domains of the neighboring molecules in the structure.

### The cleavage reaction center

**Figure 3:**
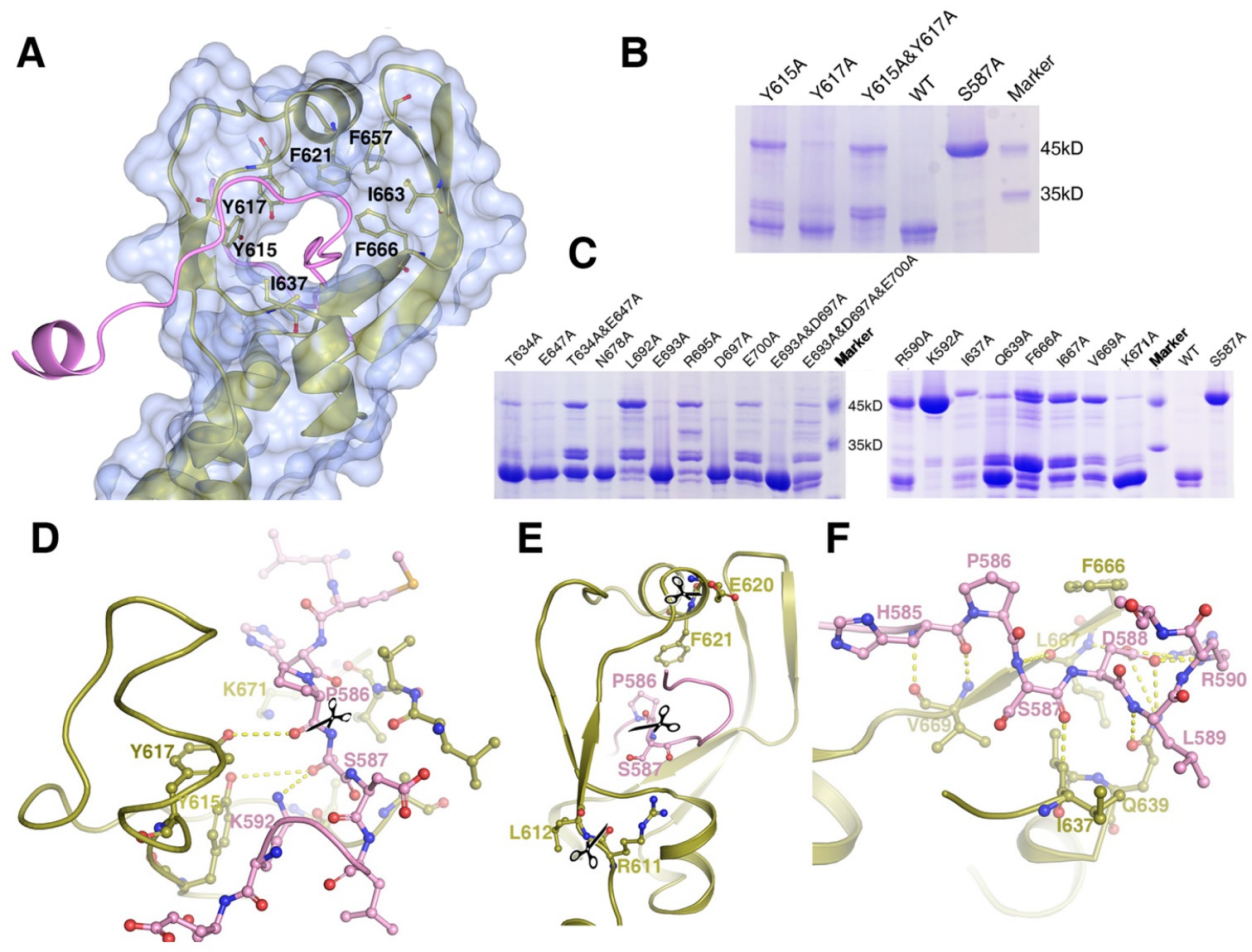
The structure of the cleavage site of the MyRF ICA domain. Depicted as red - oxygen; blue - nitrogen; gold, ice-blue and pink - carbon atoms and yellow dotted line - hydrogen bond, scissors - the scissile peptide bond. Ser587 has been modeled into the structure to illustrate the proximity of this residue to the other residues nearby. **A.** The “cable cutter” structure of the MyRF cleavage reaction center. The surface and gold cartoon diagram of the β-strands of β3-β3a-β3b-β4 and β1-α2-β2 showed a “donut” shape. The “cable” with the cleavage site from the neighboring subunit was colored pink. The aromatic and hydrophobic residues Y615, Y617, F621, I637, F657, I663, and F666 surrounding the “cutting hole” were represented as sticks too. **B.** The auto-cleavage of the mutants of MyRF (Y615A, Y617A, Y615A&Y617A) was analyzed by SDS-PAGE. 6xHis-tagged MyRF and its mutants were overexpressed and pulled out using Ni-affinity beads and visualized on Coomassie blue stained SDS-PAGE. The MyRF_wt_ and MyRF_S587A_ were set as the positive and negative control, respectively. **C.** The auto-cleavage of the mutants of MyRF (R590A, K592A, T634A, I637A, Q639A, E647A, T634A&E647A, F666A, L667A, V669A, K671A, N678A, L692A, E693A, R695A, D697A, E700A, E693A&D697A, E693A&D697A&E700A) was analyzed by SDS-PAGE. 6xHis-tagged MyRF and its mutants were overexpressed and pulled out using Ni-affinity beads and visualized on Coomassie blue stained SDS-PAGE. The MyRF_wt_ and MyRF_S587A_ were set as the positive and negative control, respectively. **D.** Close-up of the interactions between the residues of Y615, Y617, K592, K671 and the cleavage site in the structure. **E.** Close-up of the two new cleavage sites showed that they were close to the original cleavage site but located on the “donut” instead of “cable” in the structure. **F.** Close-up of the interactions between the residues of R590, I637, Q639, F666, L667, V669 and the cleavage site in the structure.

Mass Spectrometry analysis of the expressed MyRF fragment (residues 351-717) revealed that the protein was cleaved between P586 and S587, consistent with previous studies. The “cable” with the cleavage site threaded through the “donut” formed by six β-strands, similar to a cable cutter. The “cutting hole” was surrounded by aromatic and hydrophobic residues (Y615, Y617, F621, I637, F657, I663, and F666) to create a hydrophobic environment for the enzymatic reactions (Figure 3A). To identify the key residues involved in the autocatalytic self-cleavage, a series of residues close to the cleavage site were mutated to alanine. The results showed that proteins with S587A or K592A were not cleaved at all, whereas proteins with most of the other mutations were partially cleaved (Figure 3B, 3C). An analysis of the sequences and structures revealed that the residues S587 and K592 of MyRF were equivalent to residues S911 and K916 of endoNF, respectively. These two residues, which formed a serine-lysine catalytic dyad as the reaction center, were indispensable for auto-cleavage as reported previously 9, 19(Figure 3D). In the structure of MyRF, all residues in the stalk were separated from the cleavage reaction center, and no direct interaction was found. Additionally, residue K671 from the top of the α4 helix was close to the cleavage reaction center in the structure and appeared to have a function similar to that of residue R897 in the endoNF triple-β-helix, which played crucial roles in autocleavage. Unexpectedly, K671 was not shown to be important for autocleavage because cleavage was not inhibited by the substitution with the alanine (Figure 3C, D). No other basic residues were found close to the reaction center in the structure. Instead, the aromatic residues Y615 and Y617 were close to the cleavage site in the structure. Mutation analysis showed that both of these residues could partially block the cleavage, although Y615 showed stronger effects. When both of the residues were mutated to alanine, cleavage at P586-S587 was completely inhibited, and two other new cleavage sites were generated (Figure 3B). Mass spectrometry analysis showed the locations of these two new sites at residues R611-L612 and E620-F621 (Figure S3, S4). These two new sites appeared in mutant R590A, I637A, Q639A, F666A, I667A, and V669A proteins (Figure 3C). Although all of these residues were conserved in the MyRF, the mutants did not completely block autocleavage. In the protein structure, these two new cut sites were located close to the cleavage site, but on the “cutter hole” instead of the “cable” (Figure 3E). All of these mutated residues were involved in holding the residues related to cleavage in place on the “cable” to ensure the efficacy and specificity of the reactions (Figure 3F). No studies have shown that proper folding of the DBD and triple-β-helix are prerequisites for the MyRF autoproteolytic reaction; however, trimerization of the ICA domain itself is required.

### C terminal triple α-helical coiled-coil

**Figure 4:**
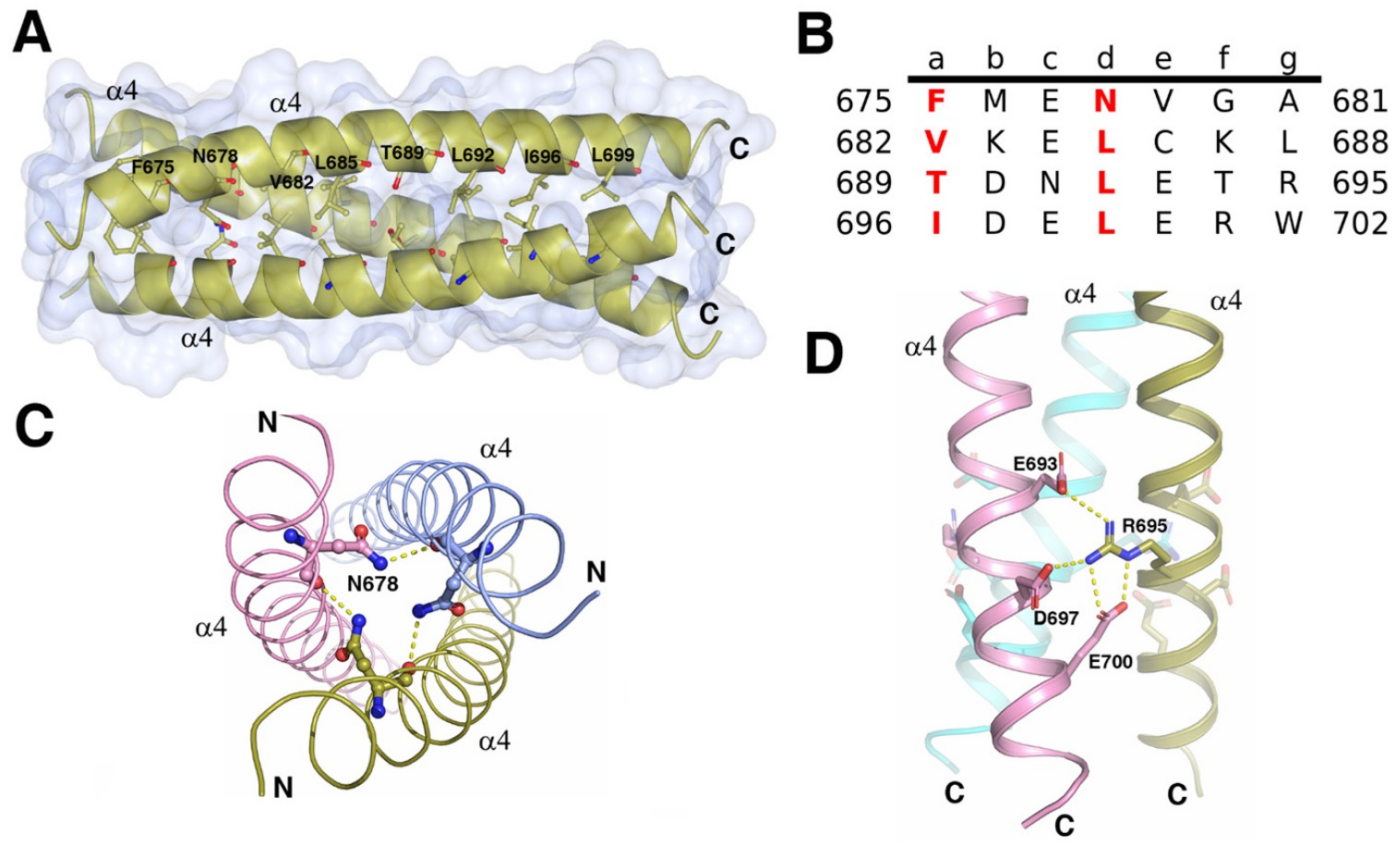
The structure of the C terminal triple α-helical coiled-coil. **A.** The crystal structure of the C terminal triple α-helical coiled-coil (N670-K705) is shown as a gold cartoon with a surface. The residues at the positions **a** and **d** of coiled-coil are represented as sticks. Depicted as red - oxygen; blue - nitrogen; gold, ice-blue and pink -carbon atoms and yellow dotted line - hydrogen bond or salt bridge.** B.** The characteristic heptad repeats of the coiled-coil domain in A. Residue number is given on both sides of the sequence. Letters **a**-**g** designate the position of the amino acids within the heptad. **C.** Three N678 from each helix may interact with each other. **D.** The R695 interacts with the E693, D697, and E700 from the neighboring helix forming a network of the interhelix salt bridges.

Trimerization of the ICA domains is mainly attributed to the triple α-helical coiled-coil, which consists of three parallel C-terminal α4 helices from three subunits (Figure 4A). Each helix buried 689Å^2^ (18% of the total accessible surface), and the total buried surface area was 4136Å^2^ (56% of the total surface area) of the trimer. The free energy of dissociation calculated using PISA was 25 kcal/mol. The sequence of each helix contained four heptad repeats (**abcdefg**)_n_ with the four “**a**” positions containing Phe, Val, Thr, and Ile and the four “**d**” positions containing one Asn and three Leu residues (Figure 4B). The hydrophobic β-branched residues Val and Ile at the “**a**” position favored the trimerization of the protein in the helices of coiled-coils 20. In addition to the force produced by burial of the hydrophobic face of the leucine zipper in the coiled-coil core, the R695 at “g” position formed interhelix salt bridges with E693, E700, and D697 at “**e**” or “**b**” positions (Figure 4D). Mutations of the residues critical for the formation of the triple α-helical coiled-coil can affect the trimerization of ICA domain and autocleavage of MyRF. Without cleavage, the transcription factor MyRF cannot be released from the ER and enter the nucleus to regulate the expression of its target genes. Single mutation of Leu at “**d**” position (L685, L692, L699) or Ile at “**a**” position (I696) was found to block the trimerization and cleavage of MyRF 7. Mutation of R695 has been shown to be related to the birth defects owing to the loss of function of MyRF 14, 21. Our results also showed that the single amino acid mutation of L692 or R695 to alanine blocked the cleavage of MyRF (Figure 3C). Among the residues interacting with R695, E700 is more important than E693 and D697 since the protein with the mutation of E700A was cleaved partially due to the destablization of the ICA trimer while the protein with the mutations of E693A and D697A had almost no effect on the cleavage (Figure 3C). The central “**d**” position asparagine (N678 of MyRF) seemed to form the buried hydrogen bonds in the core of the structure, as shown in Figure 4C, but mutation of N678 to alanine had no effect on the cleavage of MyRF in the expression of *E. coli.* (Figure 3C), which implied that it was not important to the formation of Myrf trimer.

### The triple α-helical coiled-coil was strengthened by α1 and α3/α3a helices

**Figure 5:**
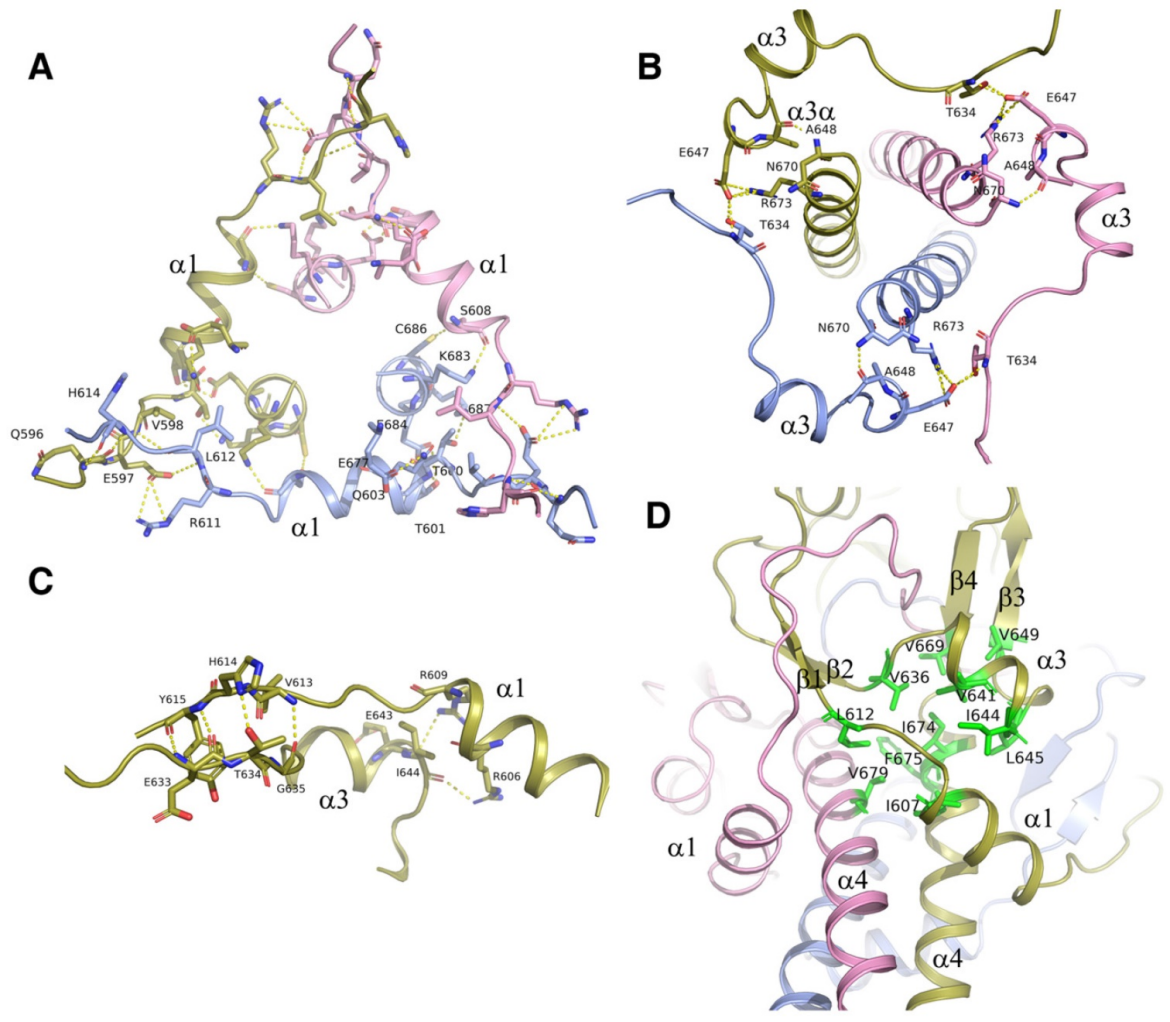
The triple α-helical coiled-coil was strengthened by surrounding structures. **A.** Three α1 helices with its flanking sequences from each subunit of the trimer interact with each other by connecting head to tail to form a triangle collar limiting the triple-helical coiled-coil in the middle. **B.** Three α3_/_α3a helices with its flanking sequences from each subunit of the trimer interact with each other by connecting head to tail to form a circular collar limiting the triple-helical coiled-coil in the middle. **C.** The interactions between α1 and α3 helix. The residues at the upstream flanking sequences of α3 helix and the residues at the downstream flanking sequences of α1 helix which are involve in the formation of hydrogen bonds and salt bridges are represented as sticks. **D.** The hydrophobic core formed at the interface of α1, α3_,_ and α4 helix with the hydrophobic residues I607, L612, V636, V641, I644, L645, V649, V669, I674, and V679 were represented as sticks and colored in green.

Structural analysis showed that the intermolecular interactions among the α1 helix, α3/α3a helix, their flanking sequences, as well as the α4 helix, also contributed to the trimerization of ICA domain. Via these interactions, the ICA trimer was greatly strengthened, demonstrating an increase in the free energy of dissociation (from 25 kcal/mol to 76 kcal/mol). Interactions among the three α1 helices of the different subunits were represented as the H-bonds and the salt bridges formed between Q596, E597, and V598 at the upstream flanking sequences of α1 helix and H614, R611 and L612 at the downstream flanking sequences of the neighboring α1 helix (Figure 5A). With these intermolecular interactions, three α1 helices from different subunits formed a trianglar collar structure outside of the triple coiled-coil by connecting head to tail to each other. Among the residues involved in these interactions, Q596, which is located upstream of the α1 helix has been reported to be related to Swyer syndrome, a disease related to the female genitalia 14.

The three α3/α3a helices also formed a circular collar structure outside of triple coiled-coil, with the hydrogen bond of E647 at α3a helix and T634 in the upstream flanking sequences of the neighboring α3 helix (Figure 5B). These collar structures strengthened the trimerization of the ICA domain by holding the triple coiled-coil in the center of the trimer. Every helix in the collar structures interacted with the central α4 helix. Residues A648 and E647 in α3a helix formed hydrogen bonds and salt bridges with the residues of N670 and R673 in the C-terminal α4 helix (Figure 5A). In the structure, the interactions were observed between α1 and α3 helices too (Figure 5C). Our results showed that the protein with the double mutation of T634A and E647A was cleaved partially due to destabilization of the ICA trimer, although each single mutation of them has little effect (Figure 3C).

In addition to hydrogen bonds and salt bridges, hydrophobic interactions also played critical roles in the trimerization of the ICA domain. The hydrophobic residues I607, L612, V636, V641, I644, L645, V649, V669, I674, and V679 at the interface of the α1, α3, and α4 helices clustered together within the protein to form a hydrophobic core, thereby stabilizing the folded state (Figure 5D). No water was present in this structural core. Notably, changes in the residues in the hydrophobic core may affect the stabilization of the ICA trimer and then alter the transcriptional function of MyRF. Moreover, mutation of V679 was recently reported to be related to birth defects caused by loss of function of MyRF 21.

## Discussion

Recent studies have identified a novel type of the membrane transcription factor, that is, MyRF. The cleavage mechanism of this membrane transcription factor was found to be similar to that of well-characterized phage tailspike proteins, which were processed via the intramolecular chaperone autocleavage (ICA) domain. In phage, the ICA domain has two functions, i.e., an intramolecular chaperone function to facilitate correct folding of the upstream triple-β-helix to generate a stable trimer and an autocatalytic self-cleavage function to release itself and leave the mature protein on the phage, trapped in a kinetically stable state. Previous studies have shown that these two functions of the ICA domain were also required for MyRF activation via trimerization, enabling the formation of a functional transcription factor, and autocleavage, releasing the protein from the ER membrane 7, 11, 19, 22.

**Figure 6:**
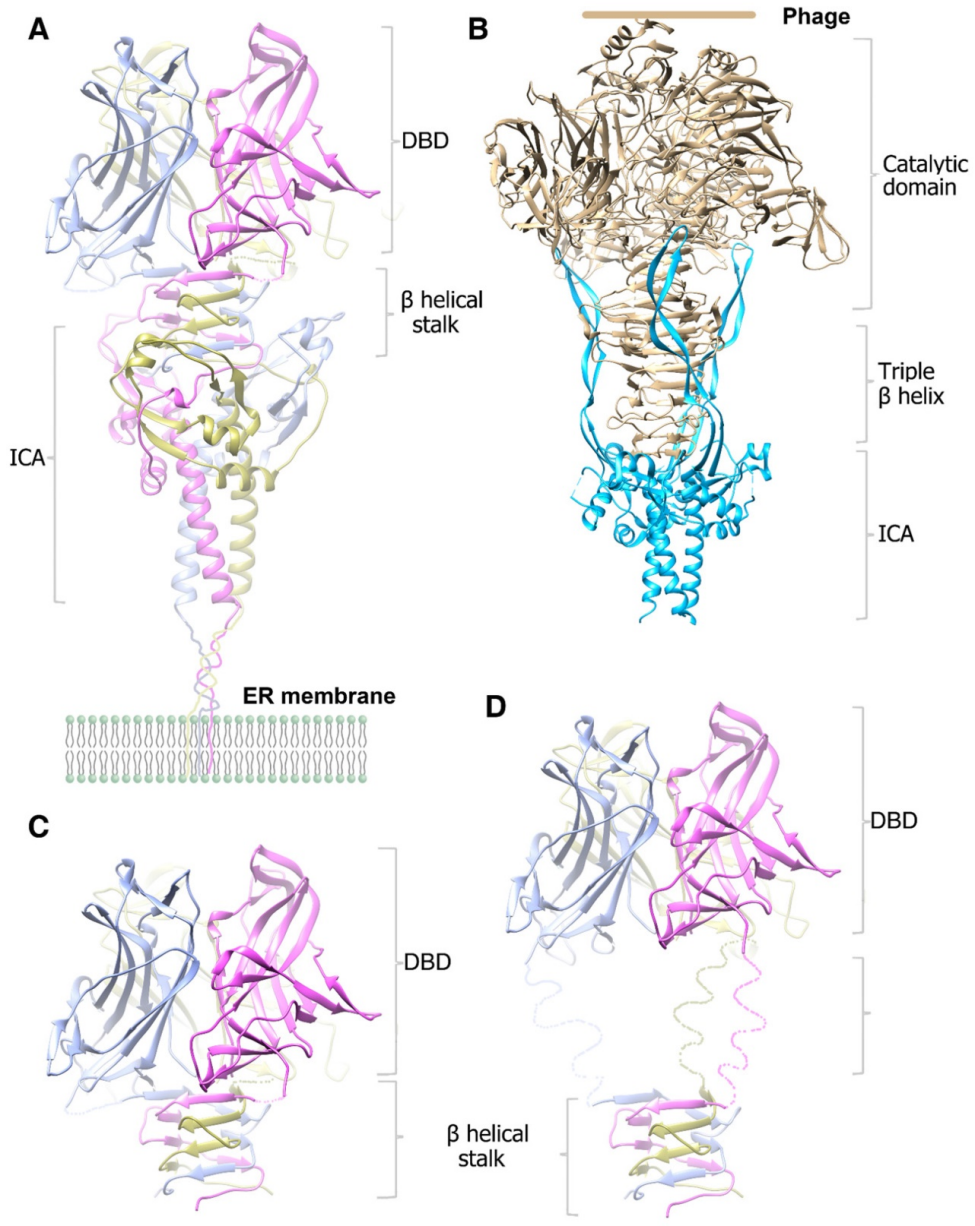
Cartoon diagram of the modeled molecular models of the homotrimer structures. **A.** The modeled pre-cleavage homotrimer structure of the MyRF N-fragment (residues 351-717), the DBD using the previously solved structure (PDB code: 5H5P). **B.** The modeled pre-cleavage homotrimer structure of the phage endoNF, using the structures of (PDB code: 3GW6 and 1V0E). **C.** The modeled cleaved homotrimer structure of the MyRF transcription factor shows the β-helical stalk helps to stabilize the DBD trimer. **D.** The modeled cleaved homotrimer structure of the MrfA transcription factor shows a long disorder linker between the DBD and β-helical stalk.

In this study, we initially wanted to obtain the structure of the precleavaged MyRF cytoplasmic core fragment containing both DBD and ICA domains by expressing the noncleavable protein MyRF_S587A_. Unexpectedly, the protein was split into two fragments at the front of the autocleavage site during crystallization, resulting in the production of DBD crystals and the stalk-ICA domain crystals. We have already reported the structure of MyRF DBD; therefore, in this study, we focused on the structure of the MyRF ICA domain with its upstream β-helical stalk. This structure revealed the molecular mechanisms underlying the homotrimerization and the autocleavage of MyRF. Moreover, the structure also elucidated the molecular differences from its correspondents in phages (Figure 6A, 6B). The most prominent difference was that the β3-β4 strands, which formed a short “cable cutter” with β1-β2 strands, and showed only weak interactions with the upstream sequence in MyRF. Another major difference was the stalk between the upstream functional domain and the ICA domain. The stalk of the MyRF mainly consisted of β-helix and formed a structure similar to the triple-β-helix in phages. This is the first structure of the mammalian protein with a triple β-helix. It required the ICA domain to trimerize like phage proteins, however, the stalk of MyRF only partially intertwined and did not form a typical fully intertwined triple-β-helix. Our analysis demonstrated that this structure was less stable than the typical triple-β-helix. There were also some differences in the cleavage reaction centers, although the cleavage mechanisms were based on the serine-lysine dyad. Residues of the triple-β-helix of endoNF participated in the cleavage reaction, and the neighboring Arg897 in triple-β-helix played a key role in the reaction; therefore, the cleavage can only be triggered by correct folding of the triple-β-helix. In contrast, few interactions were observed between the MyRF ICA domain and its upstream neighboring β-helix, and no stalk residues were found to participate in the cleavage reaction. Accordingly, cleavage of the MyRF ICA domain appeared to be independent of the folding of its upstream sequence. The finding that the mutation G575R disturbed the trimerization of the β-helical stalk, but did not affect the autocleavage 19 was consistent with this observation. Activation of MyRF cleavage may be related to the presence of some auxiliary factors on the ER membrane, as observed for other membrane proteins.

After cleavage, the ICA domain of endoNF was released from the mature protein on the phage and left a very stable stalk that was resistant to heat, sodium dodecyl sulfate (SDS), and proteases, thereby facilitating antibacterial effects (Figure 6B). In comparison, MyRF released the transcription factor from the ER membrane and maintained the ICA domain on the membrane after autoproteolysis. The mature MyRF protein (DBD with the β-helix stalk) did not show resistance to heat, SDS, or proteases *in vitro*, consistent with its role as a transcription factor.

The MyRF DBD domain itself can form an unstable homotrimer 23-25, but it was not functional when overexpressed in the cells 7. Trimerization of DBD requires stabilization by the β-helical stalk because the overexpressed MyRF DBD harboring the β-helical stalk and ICA domain was functional, whereas the single residue mutation G575R at the stalk disturbed the trimerization of the β-helix stalk, thereby blocking function 19. DBD would form a stable trimer through the triple-β-helical stalk. Indeed, the noncleavable MyRF_S587A_ protein was split into two fragments at the site between the DBD and β-helical stalk during crystallization, implying that the trimeric transcription factor released from the ER membrane may be unstable *in vitro*, but should be stable *in vivo* as a functional transcription factor (Figure 6C). The triple-β-helical stalk is the key to assure the formation of functional trimeric transcription factor. In MrfA from molt prestalk cell (Dictyostelium), there is a long, disorder linker between the DBD and triple-β-helical stalk (Figure 6D). The presence of this disorder linker suggests that the DBD may maintain some flexibility under the restraint of the triple-β-helical stalk.

The trimerization of the ICA domain not only mediated correct folding of the β-helix but was also a prerequisite for the autoproteolytical reaction of MyRF. Structure analysis demonstrated that the three ICA domain C-terminal α4 helices formed a triple α-helical coiled-coil, which became the core of the ICA trimer. Furthermore, trimerization of the ICA domain was found to be strengthened by two collar structures formed by the α1 and α3 helices with their flanking sequences around the triple helix bundle.

Recent studies have found many mutants of MyRF that are associated with various diseases 15-18. These mutations can be divided into two groups, depending on their locations. The first group that affects the transcription function of MyRF is located in the DBD domain, such as the mutations F387S and G435R, resulting in congenital diaphragmatic hernia 14; the mutation Q403R, resulting in MYRF-related mild encephalopathy with reversible myelin vacuolization (MMERV) 26; and the mutation Q443P, resulting in disorders of sex development (DSDs) 27. The second group involves trimerization-related mutations that are mainly located in the ICA domain or the triple β-helical stalk. Disruption of the trimerization of the ICA domain blocks autocleavage and fails to mediate the proper folding of the triple-β-helix, ultimately leading to failure of functional DBD trimer production, such as the mutations V679A and R695H in the C-terminal α helix, resulting in congenital diaphragmatic hernia 14.

In summary, our findings on the structure of MyRF ICA domain with its upstream β-helical stalk provided an insight into the molecular mechanisms of the MyRF trimerization and the auto-cleavage. Although the stucture in this paper revealed the intrinsic properties of MyRF and provided the hints and explanations in the molecular level to some abnormal variations found in clinic, the actural roles of the protein in cells need more studies. In addition, the recent finding that the ER-associated transmembrane protein TMEM98 inhibits MyRF self-cleavage 28 implicates that the self-cleavage of MyRF can be regulated by other proteins. The molecular mechanism of the regulation of MyRF self-cleavage requires further studies too.

## Experimental procedures

### Constructs generation

The mouse MyRF DNA fragment (residues 351-717) was amplified from a full-length gene clone (NP_001028653.1) by standard PCR. The amplified products were then cloned into a modified pRSFDuet-1 expression vector (Novagen) with an N-terminus 6xHis tag to facilitate purification. Point mutations (R590A, K592A, Y615A, Y617A, Y615A&Y617A, T634A, I637A, Q639A, E647A, T634A&E647A, F666A, L667A, V669A, K671A, N678A, L692A, E693A, R695A, D697A, E700A, E693A&D697A&E700A) were performed by the QuickChange protocol (Stratagene). All constructs were verified by DNA sequencing.

### Protein expression and purification

The protein containing both DBD and ICA domains was obtained by expression of MyRF_S587A_ (residues 351-717) in BL21 (DE3) *Escherichia coli* (Novagen) induced with 0.5 mM isopropyl-β-D-thiogalactopyranoside(IPTG) for 20 h at 16 °C. The cell lysate was clarified after lysis (50 mM Tris-HCl, 300 mM NaCl, pH 8.0, containing 1 mM PMSF, Sigma), sonication, and centrifugation and was incubated with Ni-affinity beads (GE Healthcare) and washed with lysis buffer. The protein was eluted by lysis buffer containing 300 mM imidazole and further separated by size exclusion chromatography on a Superdex-200 column (GE Healthcare) pre-equilibrated in a buffer with 20 mM Tris-HCl (pH 8.0), 300 mM NaCl. Peak fractions corresponding to pure protein were collected and concentrated to 20 mg/ml for crystallization.

Selenomethionine (SeMet) derived MyRF_S587A_ recombinant protein was overexpressed in the *E. coli* strain BL21(DE3) similar to the native protein. The bacterial cell culture medium was replaced by the M9 medium which contains 0.4% glucose, 2 mM magnesium sulfate, 0.1 mM calcium chloride. SeMet was supplemented along with leucine, isoleucine, and valine of a final concentration of 50 mg/L, and lysine, threonine, and phenylalanine of a final concentration of 100 mg/L before adding IPTG. The protocol for the purification of the SeMet derived recombinant protein was similar to that for the native protein, except 5 mM DTT was added in the buffer.

The expression and purification of the proteins with the other mutations were conducted according to the same protocols as for MyRF_S587A._ All samples were collected at each step and analyzed by SDS-PAGE.

### Crystallization and data collection

The crystallization trials were set with the final purified MyRF_S587A_ protein by the sitting drop vapor diffusion method mixed 1:1 with a reservoir solution. Crystals appeared in the reservoir of the buffer 1 containing 8% PEG6000, 0.1M Cacodylate, pH5.5, and the buffer 2 containing 10% PEG6000, 1M LiCl, 0.1M MES, pH 6.5 in about 1 month at 16°C and were frozen in a cryoprotectant consisting of the reservoir solution supplemented with 30% glycerol. Data were collected on the BL17U1 station of the Shanghai Synchrotron Radiation Facility (SSRF) and processed using the HKL2000 software 29.

### Structure determination and refinement

The crystals belonged to the P321 space group. The space group parameters of some of them were similar to the DBD crystal we got previously and the structure of them was solved by molecular replacement program PHASER 30 using the structure of MyRF DBD as a search model.

The structure of some other crystals with different space group parameters which can't find the solution with molecular replacement were solved through single isomorphous replacement including anomalous scattering (SIRAS) using a selenomethionine (SeMet) derivatized protein crystal by PHENIX AutoSol program 31.

Model building and iterative refinement were performed with the COOT and PHENIX refinement programs 31, 32. The orientations of the amino acid side chains and bound water molecules were modeled based on 2F_obs_-F_calc_ and F_obs_-F_calc_ difference Fourier maps. Detailed data collection and refinement statistics were listed in table 1. The model figures were generated with PyMol, CCP4mg 33, and Chimera 34. The interactions were analyzed with PyMol and PISA 35, 36.

### Mass spectrometry

The protein samples were expressed and purified with the method in the procedure and the interested peak fractions from gel filtration were collected and concentrated. The following chromatographic separation of these samples was performed by a UPLC system (Shimadzu UPLC LC-30DA, Japan) equipped with an ACE C4 column (2.1 mm x 100 mm, 5 μm). An elution gradient was used by mixing mobile phase A (0.1% formic acid in water) with solvent B (0.1% formic acid in acetonitrile), as follows: gradient elution with 2% B from 0 to 1 min; increasing to 90% B from 1 to 7 min; elution with 90% B from 7 to 7.5 min; decreasing to 2 % B from 7.5 to 7.6 min; elution with 2% B from 7.6 to 8.0 min. Mass spectrometry was performed with a hybrid triple quadrupole time-of-flight mass spectrometer (AB SCIEX Triple TOF™ 4600 equipped with a Duo Spray Ion Source, Foster City, CA, USA). Three microliters of the samples were injected. The proteins eluted from the column at a flow rate of 0.3 L/min were introduced into the source of the mass spectrometer on-line. The ion spray voltage is operated at 5500 V. The turbo spray temperature (TEM) was maintained at 380℃. The samples were analyzed by the Peakview software in which each full TOF-MS/MS scan was acquired at a TOF resolution of 1,000 and followed by 105-2500 input m/z scans. The output resolution was set to 0.25 Daltons.

**Data availability:** The structure presented in this paper has been deposited in the Protein Data Bank (PDB) with the codes: 7DC3. All remaining data are contained within the article.

## Supplementary Material

Supplementary figures.

## Figures and Tables

**Table 1:** Data collection and refinement statistics

	MyRF ICA domain
**Data collection**	
Wavelength	0.9792
Resolution (Å)	38.22 - 2.40 (2.486 - 2.400)*
Space group	P321
Cell dimensions	
*a*, *b*, *c* (Å)	78.7 78.7 138.5
α, β, γ (º)	90.0 90.0 120
Total reflections	364924 (26011) *
Unique reflections	20050 (1965) *
Multiplicity	18.2 (13.2) *
Completeness (%)	96.00 (69.90) *
Mean I/sigma(I)	16.78 (2.00) *
Wilson B-factor	30.67
R-merge	0.7467 (5.523) *
CC ½*	0.971 (0.0291) *
**Refinement**	
Resolution (Å)	38.22 - 2.40
*R*_work_ / *R*_free_	0.217/0.268
Reflections used in refinement (working/test)	19263/1375
No. non-hydrogen atoms	3468
macromolecules	3383
Water	85
*B*-factors	59.06
macromolecules	58.39
Water	42.72
Clashscore	2.50
R.m.s. deviations	
Bond lengths (Å)	0.002
Bond angles (º)	0.47
Ramachandran plot	
Favored regions (%)	98.34
Allowed regions (%)	1.43
Outliers (%)	0.24

* Values in parentheses are statistics for the highest resolution shell. One crystal was used for this data set.
