# Supplementary material for: Crystal structure of the MyRF ICA domain with its upstream β-helical stalk reveals the molecular mechanisms underlying its trimerization and self-cleavage
Source: Int J Biol Sci. 2021 Jul 13;17(11):2931–43. doi: 10.7150/ijbs.57673 (PMC8326128; doi:10.7150/ijbs.57673)
Supplement: Supplementary file 1 — Supplementary figures. [file ijbsv17p2931s1.pdf]

Supplementary Materials:

Crystal structure of the MyRF ICA domain with its upstream β-helical stalk reveals the molecular mechanisms underlying its trimerization and self-cleavage

Pei Wu[1, 3], Xiangkai Zhen[1#], Bowen Li[1], Qian Yu[1], Xiaochen Huang[1], Ning Shi[1, 2*]

[*]Corresponding Author: Ning Shi
E-mail: shining@fjirsm.ac.cn or ningshi2000@hotmail.com

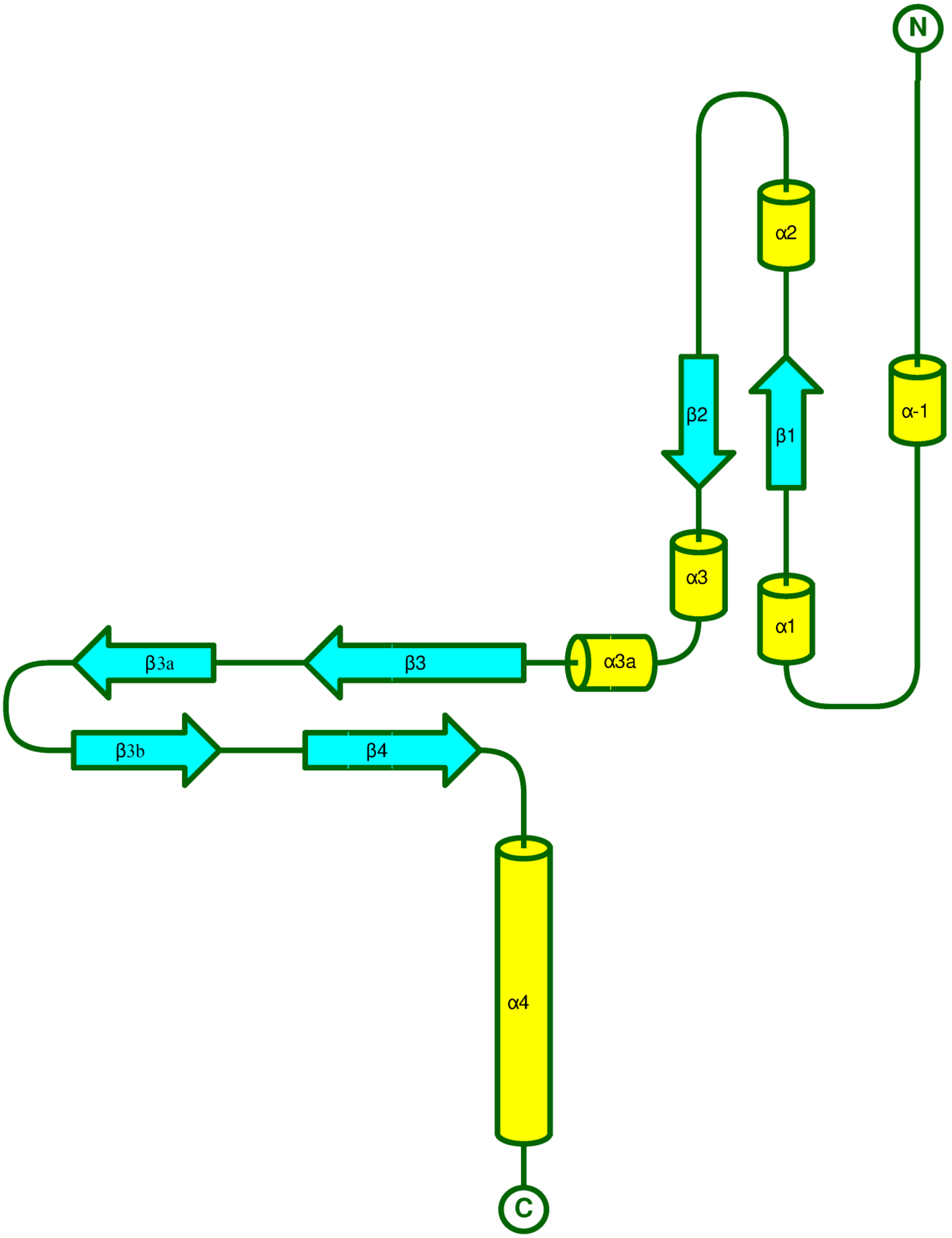

Figure S1. Topology diagram of the MyRF ICA domain.
The topology diagram gives an overview of the consecutive secondary structure elements found in the MyRF ICA domain.

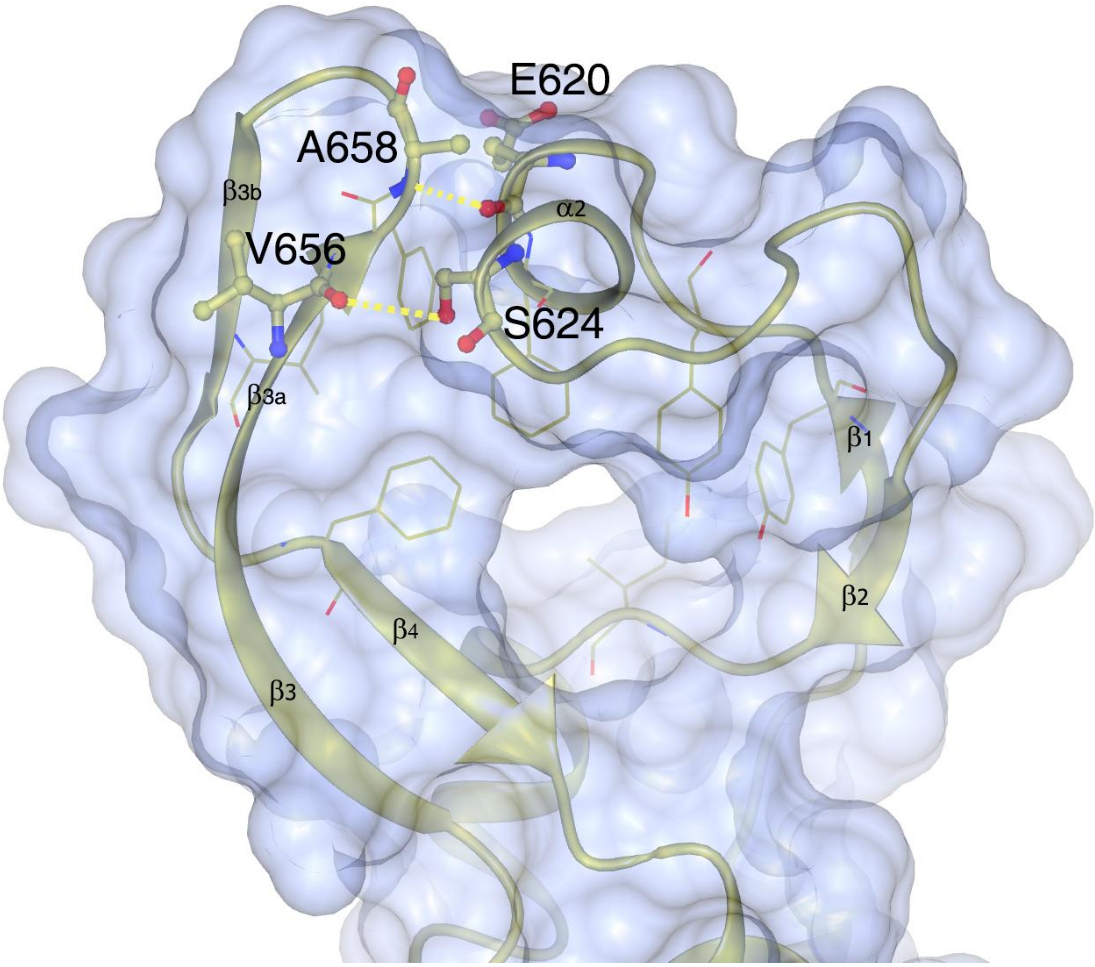

Figure S2. The hydrogen bonds were formed at the top of the “donut” which was formed of the β3-β4 and β1-β2 strands in MyRF.

E620, S624 of the helix α2 （between the stands of β1 and β2） and A658, V656 of the loop (between the stands of β3a and β3b) were represented as sticks. The hydrogen bonds were formed between them and were showed as the yellow lines.

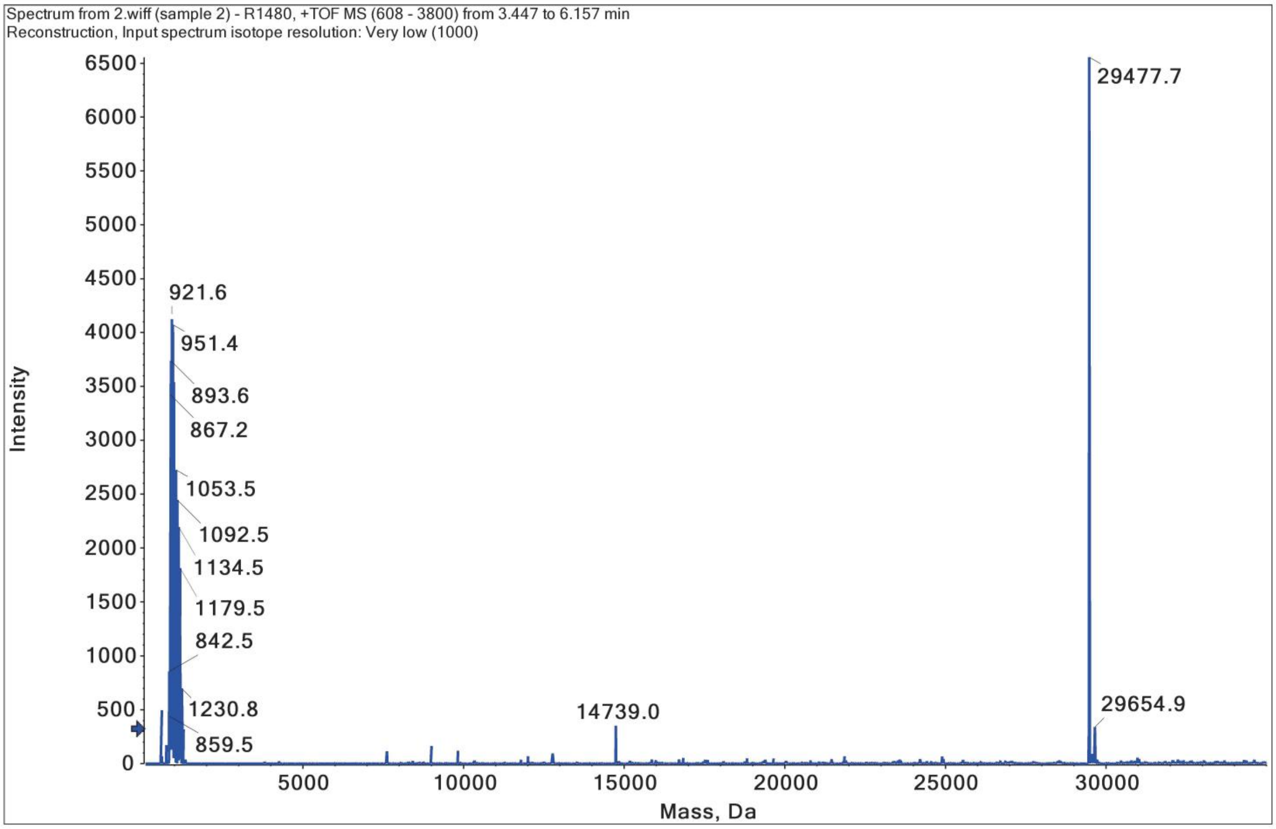

2019/11/13 15:47:16

Figure S3. Mass Spectrometric Analysis of $MyRF_{wt}$ (351-717) product
The result of MALDI-TOF-Mass spectrometry shows the molecular weight of MyRF DBD after auto-cleavage which matches the sequence 351-586 of MyRF (plus his tag and the linker residues) with calculated MW 29, 477 Dalton.

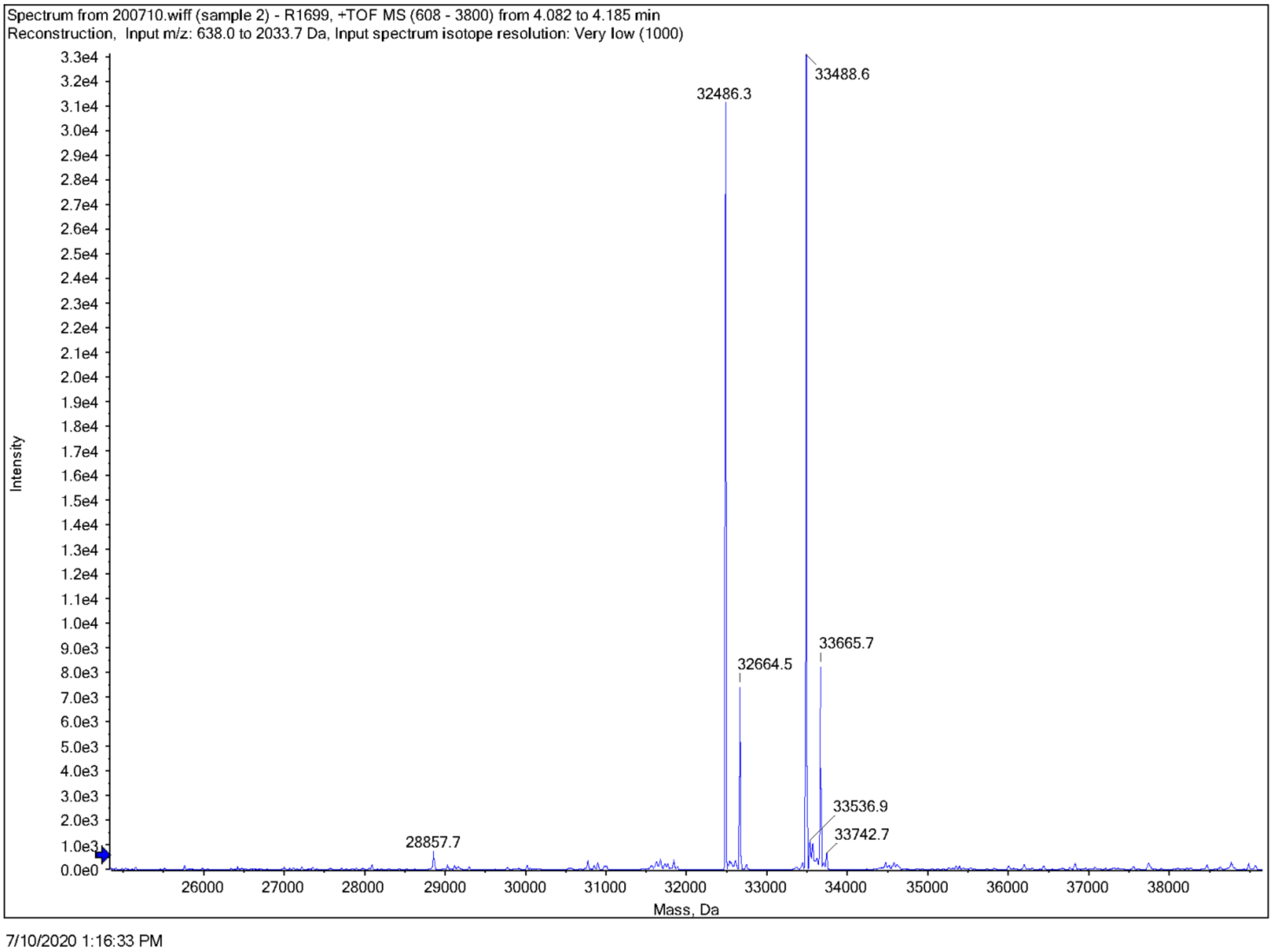

Figure S4. Mass Spectrometric Analysis of MyRFY$_{Y615A\&Y617A}$ product
The result of MALDI-TOF-Mass spectrometry shows the molecular weight of MyRF DBD after auto-cleavage which matches the sequence 351-611 and 351-620 of MyRF (plus his tag and the linker residues) with calculated MW 32,486 Dalton and 33,488 Dalton.
